# Inhibition of Anaplastic Lymphoma Kinase (Alk) as Therapeutic Target to Improve Brain Function in Neurofibromatosis Type 1 (Nf1)

**DOI:** 10.3390/cancers15184579

**Published:** 2023-09-15

**Authors:** Joseph B. Weiss, Jacob Raber

**Affiliations:** 1 Cardiovascular Institute and Warren Alpert School of Medicine at Brown University, Providence, RI 02840, USA; 2 Departments of Behavioral Neuroscience, Neurology, and Radiation Medicine, Division of Neuroscience, ONPRC, Oregon Health & Science University, Portland, OR 97239, USA

**Keywords:** water maze, spatial learning and memory, cognitive flexibility, fear conditioning, circadian activity levels, sleep, extinction of memory

## Abstract

**Simple Summary:**

Neurofibromatosis type 1 (Nf1) is a genetically determined neurodevelopmental disorder and tumor syndrome with an incidence of approximately 1/3000 live births. It is caused by loss of function mutations in the neurofibromin gene (*Nf1*) and is estimated to affect 100,000 people in the US. Approximately 50% of the patients inherited the condition in an autosomal dominant pattern; the remaining 50% are the result of de novo mutations. Behavioral alterations and cognitive deficits have been found in 50–70% of children with Nf1. These behavioral alterations and cognitive deficits include specific problems with attention, visual perception, language, learning, attention, and executive function. Inhibition of the anaplastic lymphoma kinase (Alk), a kinase which is negatively regulated by neurofibromin, allows for testing the hypothesis that this inhibition may be therapeutically beneficial in Nf1. In this review, we discuss this area of research and directions for the development of alternative therapeutic strategies to inhibit Alk.

**Abstract:**

Neurofibromatosis type 1 (Nf1) is a neurodevelopmental disorder and tumor syndrome caused by loss of function mutations in the neurofibromin gene (*Nf1*) and is estimated to affect 100,000 people in the US. Behavioral alterations and cognitive deficits have been found in 50–70% of children with Nf1 and include specific problems with attention, visual perception, language, learning, attention, and executive function. These behavioral alterations and cognitive deficits are observed in the absence of tumors or macroscopic structural abnormalities in the central nervous system. No effective treatments for the behavioral and cognitive disabilities of Nf1 exist. Inhibition of the anaplastic lymphoma kinase (Alk), a kinase which is negatively regulated by neurofibromin, allows for testing the hypothesis that this inhibition may be therapeutically beneficial in Nf1. In this review, we discuss this area of research and directions for the development of alternative therapeutic strategies to inhibit Alk. Even if the incidence of adverse reactions of currently available Alk inhibitors was reduced to half the dose, we anticipate that a long-term treatment would pose challenges for efficacy, safety, and tolerability. Therefore, future efforts are warranted to investigate alternative, potentially less toxic and more specific strategies to inhibit Alk function.

## 1. Introduction

### Neurofibromin Function in Neurofibromatosis and Its Link to Anaplastic Lymphoma Kinase

**Figure 1:**
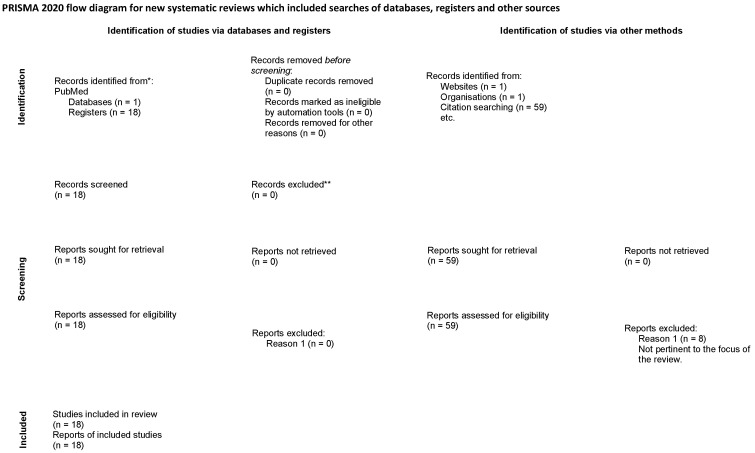
PRISMA flow diagram illustrating the review searches and reviews included as part of this paper. Template From [1].

Relatively little is published about Nf1 and Alk interaction. A PubMed search of Nf1 and anaplastic lymphoma kinase revealed only 18 references, and all results were included in this review. In addition, we carried out a Google search; 59 references were revealed and all the results pertinent to the topic were included in this review, as well (please see the Preferred Reporting Items for Systematic Reviews and Meta-Analyses (PRISMA) (Figure 1)). Reports not retrieved (*n* = 0).

Neurofibromatosis type 1 (Nf1) is a genetically determined neurodevelopmental disorder and tumor syndrome with an incidence of approximately 1/3000 live births. It is caused by loss of function mutations in the neurofibromin gene (*Nf1*) and is estimated to affect 100,000 people in the US. Approximately 50% of the patients inherit the condition in an autosomal dominant pattern; the remaining 50% are the result of de novo mutations. Nf1 is a tumor predisposition syndrome [2,3,4,5,6,7,8,9,10]. Behavioral alterations and cognitive and deficits have been found in 50–70% of children with *Nf1*. These behavioral alterations and cognitive deficits include specific problems with attention, visual perception, language, learning, attention, and executive function. They are observed in the absence of tumors or macroscopic structural abnormalities in the central nervous system [11,12,13,14,15,16]. No effective treatments for the behavioral and cognitive disabilities of Nf1 exist.

Positional cloning and sequence analysis of the *Nf1* gene revealed that it is a negative regulator of the small GTPase, Ras, a proto-oncogene and activator of the mitogen-activated protein kinase (MAP kinase) cascade [17,18,19,20,21]. When Ras is activated, typically by a receptor tyrosine kinase, it localizes to the plasma membrane and binds to GTP. Hydrolysis of GTP to GDP converts Ras from an active to an inactive form. Neurofibromin is a Ras-GTPase activating protein that catalyzes the conversion of active GTP-Ras to inactive GDP-Ras. Based on its structure, neurofibromin was predicted to be a direct negative regulator of Ras and, in turn, of the MAP kinase signal transduction cascade. This biochemical function of neurofibromin is strongly supported by the tumor suppressor phenotype of *Nf1* mutations [22]. 

Inappropriate hyperactivation of the Ras-MAP kinase cascade by mutation of *Nf1* has been confirmed in humans and murine models [23,24,25,26,27,28,29]. Pharmacologic inhibition, even transiently, of the Ras-MAP kinase mice has ameliorated several developmental phenotypes linked to *Nf1* mutation. These developmental phenotypes include abnormal cell fate determination in juvenile and adult neurogenesis and cerebellar development [30]. The cognitive impairments observed in heterozygous *Nf1* mice have also been rescued by inhibition of the Ras-MAP kinase cascade [31]. The observation that Nf1 is caused by haploinsufficiency or partial loss of function of the *Nf1* gene implies that the phenotype may be amenable to interventions that modulate the signaling pathway in which it acts.

**Figure 2:**
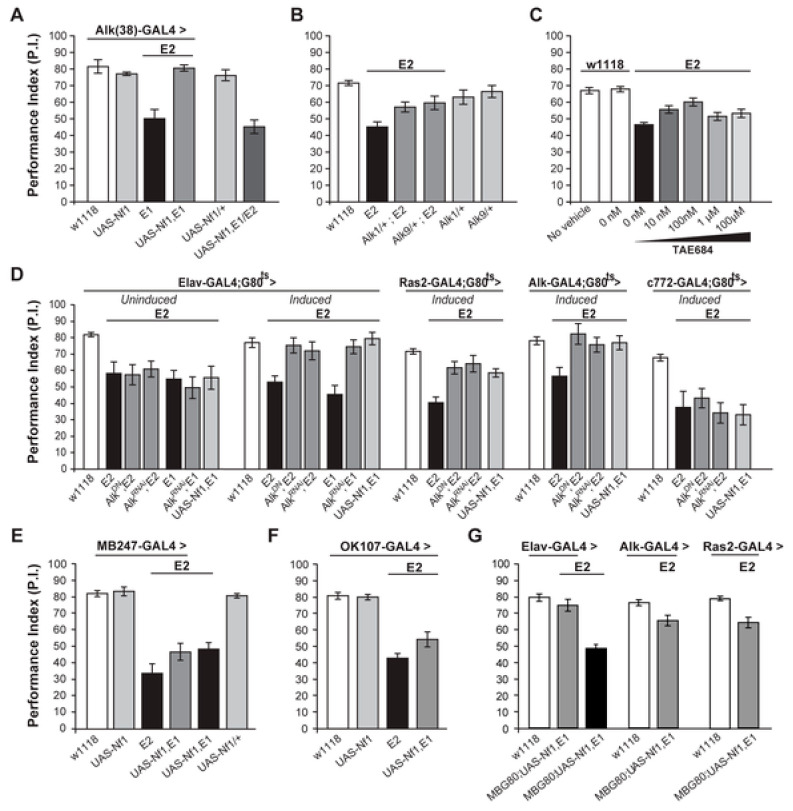
The receptor tyrosine kinase Alk controls dAlk interacts with dNf1 to regulate learning. (**A**) *Alk(38*)-Gal4 expression of UAS-*Nf1* in *Nf1*^E1/E2^ null mutants rescues learning. Heteroallelic *Nf1*^E1/E2^ mutant flies were used for ease of genetic manipulations. ANOVA indicated significant effects of genotype (F_(5,40)_ = 20.36, *p* < 0.0001, *n* ≥ 6), whereas no significant differences between *Alk(38)*-Gal4 driven UAS-*Nf1* in *Nf1*^E1/E2^ mutants and *Alk(38)*-Gal4/+ controls were found. (**B**) *Alk*^1^ or *Alk*^9^ heterozygous mutant alleles and *Nf1*^E2^ interact genetically to rescue learning. The performances of *Alk*^1^/+; *Nf1*^E2^, *Alk*^9^/+;*Nf1*^E2^ and *Nf1*^E2^ were significantly different (ANOVA: F_(5,79)_ = 8.78, *p* < 0.0001, *n* ≥ 12). (**C**) The Alk inhibitor TAE684 rescues *Nf1*^E2^ learning defects (ANOVA: F_(6,81)_ = 18.60, *p* < 0.0001, *n* ≥ 10). Planned comparisons (shown in Table S1) showed significant differences between 0 nM and 10 nM or 100 nM concentrations. (**D**) Adult induced dAlk down-regulation pan-neuronally or in Ras2- and Alk(38)- expressing cells rescues *Nf1*^E2^ learning defects. ANOVA indicated significant effects of *Nf1*^E2^ mutants expressing UAS-*Alk*^DN^ or UAS-*Alk*^RNAi^ when compared to *Nf1*^E2^ alone (F_(6,55)_ = 9.49, *p* < 0.0001, *n* ≥ 7 for *Elav*-Gal4;Gal80^ts^ (‘*Induced*’), F_(4,41)_ = 15.89, *p* < 0.0001, *n* ≥ 8 for *Ras2*-Gal4; Gal80^ts^, F_(4,38)_ = 4.15, *p* < 0.0078, *n* ≥ 6 for *Alk(38)*-Gal4;Gal80^ts^ respectively). No rescue is observed upon bearing but not expressing the transgenes pan-neuronally (‘*Uninduced*’), or using the MB-specific *c772*-Gal4;G80^ts^ driver. (**E**) Over-expression of a wild-type dNf1 transgene (UAS-*Nf1*) in the MBs using *MB247*-Gal4 fails to restore learning in *Nf1* mutants. ANOVA indicated significant effect of genotype (F_(5,46)_ = 23.31, *p* < 0.0001, *n* ≥ 7 for all genotypes). Planned pairwise comparisons indicated significant differences between *Nf1*^E2^ mutant flies harboring the *MB247*-Gal4 driver, UAS-*Nf1* transgene, or mutant flies overexpressing the wild-type dNf1 transgene in the MBs and heterozygous driver controls (*p* < 0.0001 for all comparisons). (**F**) Over-expression of a wild-type dNf1 transgene (UAS-*Nf1*) in mushroom body neurons using *OK107*-Gal4 during development fails to restore performance of *Nf1* mutants. ANOVA indicated significant effect of genotype (F_(3,33)_ = 37.72, *p* < 0.0001, *n* ≥ 8 for all genotypes). Planned pairwise comparisons indicated significant differences between *Nf1*^E2^ mutant flies harboring the *OK107*-Gal4 driver, or mutant flies overexpressing the wild-type dNf1 transgene in *OK107*-marked mushroom body neurons and heterozygous driver controls (*p* < 0.0001 for all comparisons). (**G**) Pan-neuronal expression of a wild-type dNf1 transgene (UAS-*Nf1*) or in Alk- and Ras2- expressing neurons with the exclusion of the MBS restores learning in *Nf1*^E1/E2^ mutant flies. ANOVA indicated significant effect of genotype (F_(6,66)_ = 20.13, *p* < 0.0001, *n* ≥ 8 for all genotypes). Planned pairwise comparisons indicated significant differences between *Nf1*^E1/E2^ flies harboring the MB-Gal80 (MBG80) and UAS-*Nf1* transgenes and mutant flies overexpressing the transgene pan-neuronally, as well as in Alk- positive and Ras2-positive cells excluding mushroom body neurons (*p* < 0.0001). The performance of Elav;MBG80;UAS-*Nf1*,*Nf1*^E1/E2^ flies was not statistically different from their respective heterozygous driver control indicating full rescue (*p* = 0.26), contrary to the performance of flies overexpressing the transgene in Alk- positive and Ras2- positive cells, indicating partial rescue (*p* = 0.003 and *p* = 0.0003 for *Alk(38)*-Gal4 and *Ras2*-Gal4 respectively). Error bars denote S.E.M. neurofibromin functions in Drosophila growth and learning. Based on [22].

In 2011, this potential therapeutic strategy became a reality when Gouzi et al., published a ground breaking paper establishing genetic interactions between *Drosophila Nf1* (*dNf1*) and *Drosophila Anaplastic Lymphona Kinase* (*dAlk*) [23] (Figure 2). *dAlk* encodes a receptor tyrosine kinase and activator of the Ras-Map kinase cascade. It is a homologue of the human proto-oncogene anaplastic lymphoma kinase (Alk). *dNf1* phenotypes, including regulation of body size and olfactory associative learning, were reproduced by hyperactivation of dAlk and rescued by inhibition of dAlk. Importantly, the associative learning phenotype could be rescued in adults, both pharmacologically and genetically. They also clearly established that the relevant tissue mediating the phenotypes was neuronal. The implications of these pioneering studies include a direct inhibitory relationship between dNf1 and dAlk signaling and, reciprocally, the potential for therapeutic intervention in neurofibromatosis by inhibition of human Alk [23].

The specific *dNf1*–*dAlk* interactions were independently confirmed and extended by Walker et al. [24]. In an unbiased genetic screen for dominant modifiers of the body size phenotype of *dNf1* mutations, both *dAlk* and its ligand *jeb* were identified as components of a dNf1-inhibited signaling pathway. They confirmed the interaction with respect to a *dNf1* neuromuscular junction phenotype and confirmed that the inhibitory interaction was evolutionarily conserved in a human neuroblastoma tissue culture model. Gamma aminobutyric acid (GABA) seems to be important in the cognitive phenotype. dNf1 loss results in excess GABAergic signaling in the central nervous system for associative learning mushroom body (MB) neurons, apparently suppressing learning [32]. Interestingly, in mice lacking Alk, GABA neurotransmission is enhanced in the central nucleus of the amygdala and this is potentiated after acute alcohol administration [33]. GABA neurotransmission is affected in Nf1 patients, as well. Nf1 patients have decreased GABA levels in the occipital cortex and frontal eye fields and decreased GABA-A receptor binding in the parieto-occipital cortex, midbrain, and thalamus [34]. Medial frontal levels of GABA were associated with intellectual abilities in Nf1 patients and the relationship between inhibitory control and medial frontal GABA was reversed in Nf1 patients with higher GABA levels associated with a faster response style, whereas in controls, it was related to a cautious strategy [35].

**Figure 3:**
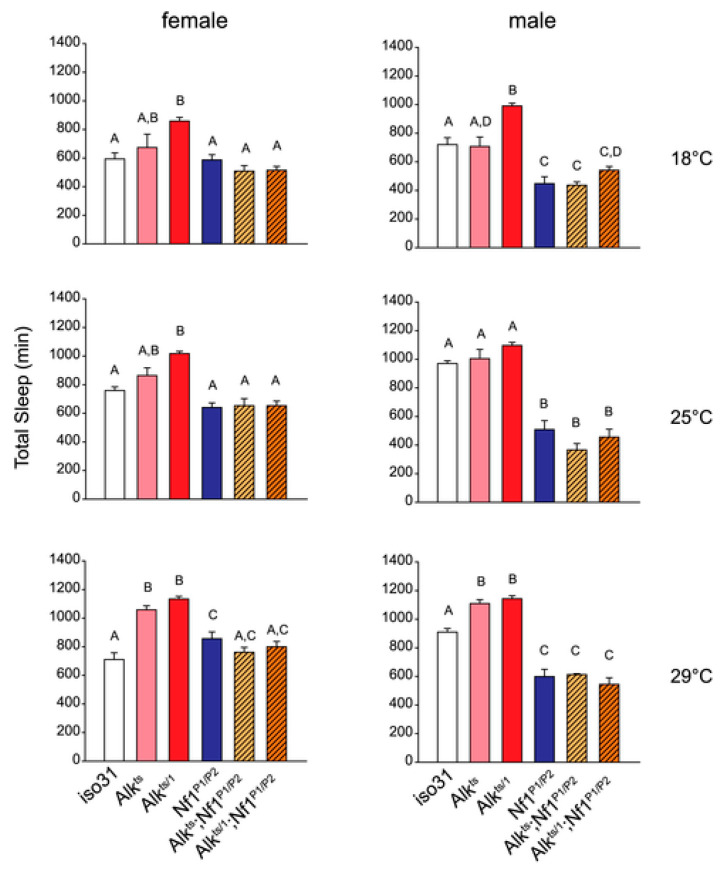
Anaplastic lymphoma kinase acts in the Drosophila mushroom body to negatively regulate sleep. *Nf1* mutations suppress the long-sleep phenotype of *Alk* mutants. Bar graphs show total sleep for *iso31* controls, *Alk* mutants, *Nf1^P1/P2^* mutants, and *Alk; Nf1* double mutants. One-way ANOVA and Mann-Whitney *post hoc* analysis was performed to compare groups in each condition. In each graph, groups with the same alphabet label on top are not statistically different from each other; groups with different labels are statistically different (*p* < 0.05). For experiments at 18 °C and 25 °C, *n* = 5–16. For 29 °C experiment, *n* = 10–37. All flies were raised at 18 °C. Activities were monitored at 18 °C, 25 °C and 29 °C in independent experiments. Based on [38].

Disordered sleep is another phenotype associated with Nf1, which is probably related to learning and memory. Pediatric patients have been documented to have problems with initiating and maintaining sleep, arousal, sleep-wake transition, and hyperhidrosis [36,37,38]. This aspect of Nf1 has not been studied extensively in humans or in model systems. Again, fundamental investigations in Drosophila uncovered an interaction between *dAlk* and *dNf1*. Bai and Sehgal studied the roles of dAlk and dNf1 in the regulation of sleep in Drosophila [39] (Figure 3). They found that *dAlk* loss of function mutations acting in the central nervous system have increased sleep and that *dNf1* mutants have reduced sleep [39]. As in the studies of Gouzi et al. [23] and Walker et al. [24], *dNf1* and *dAlk* interacted genetically in a fashion consistent with *dAlk* inhibition rescuing the *dNf1* short-sleep phenotype. Reduced dAlk signaling in the central nervous system of *dNf1* mutant flies rescued or reversed the sleep phenotype similar to the associative learning phenotype. Consistent with these results, Nf1 mice showed increased sleep fragmentation [40]. 

In humans, Alk was originally identified as an oncogene that is inappropriately activated by chromosomal translocation in anaplastic lymphomas [41]. Subsequently, it was determined to cause neuroblastoma, non-small cell lung cancer, and inflammatory myofibroblastic sarcoma [42,43,44,45,46]. It is also a likely culprit in some renal cell, breast, esophageal, colonic, glial, and thyroid cancers [47]. As a frequently mutated or dysregulated gene implicated in a variety of human tumors, Alk has been therapeutically targeted by orally active small molecule inhibitors [48,49]. First-generation Alk inhibitors have demonstrated efficacy against these tumors and are in clinical use [50]. Crizotinib, the first FDA-approved treatment for non-small cell lung cancer, is very active against the primary tumors, but has limited or no penetration into the brain. Therefore, cancer that has metastasized to the brain cannot be treated well with this compound. Newer agents such as alectinib have proven central nervous system activity and efficacy, treating metastatic disease in the brain that is resistant to treatment with crizotinib [51,52].

The pioneering studies in Drosophila are the predicate for a more specific therapeutic approach to the treatment of neurobehavioral Nf1 phenotypes in humans. They provided the rationale for experiments we performed to test the efficacy of Alk inhibition in Nf1 heterozygous mutant mice (for a review about Nf1 mouse models, see [53]). Specifically, we have tested the hypothesis that genetic and pharmacologic inhibition of Alk in a mouse model of Nf1 would ameliorate or even reverse Nf1-associated neurocognitive disabilities and disordered sleep. Confirmation of this hypothesis in mice is a necessary prelude to evaluating the therapeutic benefit of Alk inhibition in humans. It is also critical to assess and minimize toxicity, as these studies have the ultimate objective of developing a treatment for humans with neurofibromatosis. Acceptable side effects in the context of cancer are not acceptable in the context of long-term treatment of patients with neurofibromatosis.

## 2. Genetic Inhibition of Alk Rescues Learning and Sleep Behaviors in Nf1 Heterozygous Mutant Mice

Our initial studies sought to replicate in part the previously reported genetic interaction between dAlk and dNf1 in Drosophila. We employed two established models of learning and memory in mice, the Morris water maze and contextual fear learning, memory and extinction. The Morris water maze is an assay of hippocampus-dependent spatial learning and memory. It consists of training mice to locate a hidden, submerged platform in an opaque fluid and evaluation of their ability both to learn multiple locations and to recall the location after the platform has been removed in probe trials. We had previously demonstrated that Alk mutant mice showed enhanced spatial learning and memory independent of Nf1 genotype. 

**Figure 4:**
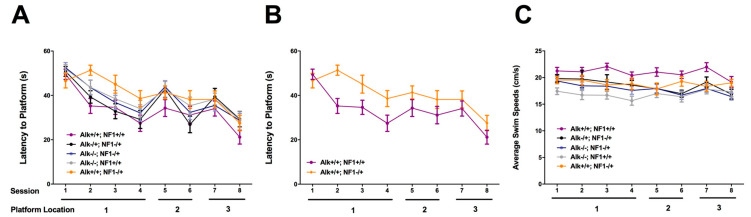
(**A**) Latency to reach the platform in the water maze. The mice were trained to locate a platform in three distinct locations during hidden platform water maze training (Hidden Location 1: sessions 1–4; Hidden Location 2: sessions 5–6; Hidden Location 3: sessions 7–8). Subsequently, the mice were trained to locate a visible platform (Visible Location: sessions 9–10). (**B**) Compared to Alk+/+; Nf1+/+ mice, Alk+/+; Nf1−/+ mice showed impairments in their ability to reach the platform (*p* = 0.048, Tukey–Kramer; *p* = 0.022, Dunnett’s multiple comparisons). Reducing Alk in the Alk−/+; Nf1−/+ and Alk−/−; Nf1−/+ mice mitigated this impairment. (**C**) Swim speeds in the water maze. Compared to Alk+/+; Nf1+/+ mice, Alk−/−; Nf1+/+ mice showed reduced swim speeds (*p* < 0.001). For training to locate the first platform location, there was an effect of Alk (*F*(2,111) = 7.456, *p* = 0.001) and a Nf1–Alk interaction (*F*(1,111) = 7.490, *p* = 0.007). For training to locate the second platform location, there was an effect of Alk (*F*(2,111) = 6.096, *p* = 0.003). For training to locate the third platform location, there was an effect of Alk (*F*(2,111) = 4.088, *p* = 0.019). Adapted based on [53].

We compared four genotypes: Alk+/+; Nf1+/+, Alk+/+; Nf1−/+, Alk−/+; Nf1−/+ and Alk−/−; Nf1−/+ for performance in the Morris water maze (Figure 4). Heterozygous Nf1 mutant mice (Alk+/+; Nf1−/+), compared to wildtype (Alk+/+; Nf1+/+), had statistically significantly greater difficulty learning to locate the hidden platform based on the time it took them to find the platform in repeated trials. This learning phenotype in Nf1 heterozygous mice was completely rescued by removal of one copy of functional Alk (Alk−/+; Nf1−/+). We also found that removal of both functional Alk genes (Alk−/−; Nf1−/+), while it improved performance in this test compared to Nf1 heterozygotes (Alk+/+; Nf1−/+), was less effective than Alk heterozygosity (Alk−/+; Nf1−/+).

We interpret these results as confirmation of an evolutionarily conserved genetic interaction between Alk and Nf1 in spatial learning, and suggestive of a balance between Alk and Nf1 activity that may have an optimal level to facilitate learning and memory.

**Figure 5:**
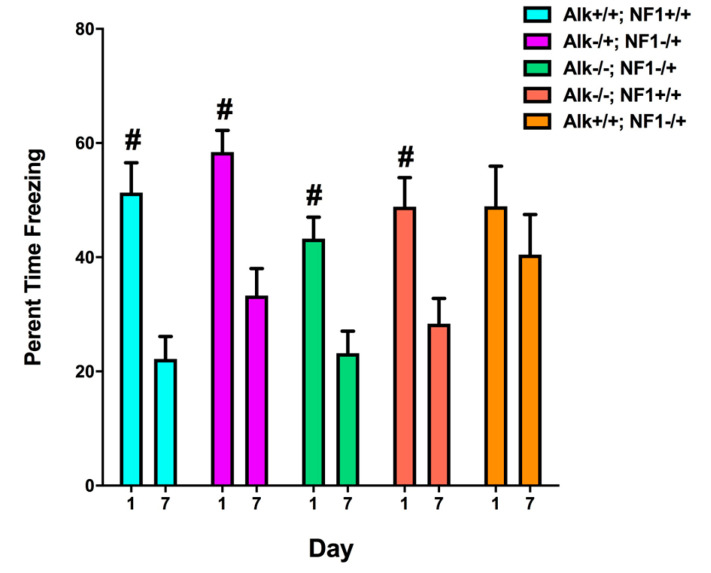
Extinction of contextual fear memory. The difference in freezing levels between days 1 and 7. The freezing levels on day 1 were significantly higher than those on day 7 in Alk+/+; Nf1+/+, Alk−/+; Nf1−/+, Alk−/−; Nf1−/+ and Alk−/−; Nf1+/+, but not in Alk+/+; Nf1−/+ mice (^#^ *p* < 0.0001, Sidak’s multiple comparisons). These data indicate a lack of extinction of contextual fear in Nf1 mice that is mitigated by reducing Alk expression. Adapted based on [54].

We next sought to test contextual fear learning and extinction (Figure 5). There was no observed genetic interaction between Alk and Nf1 with respect to this phenotype, but we observed a reduction in the response to shock (average motion) in Nf1 heterozygotes (Alk+/+; Nf1−/+) that was the same as wildtype (Alk+/+; Nf1+/+) in Nf1 heterozygotes that were also heterozygous (Alk−/+; Nf1−/+) or homozygous (Alk −/−; Nf1−/+) mutants in Alk, consistent again with a genetic interaction between Alk and Nf1. Extinction of contextual fear response showed a similar rescue of Nf1 heterozygous phenotype by reduction in Alk function. In this assay, mice are evaluated for a fear response, freezing, in the absence of an aversive stimulus over a period of a week. Nf1 heterozygotes (Alk+/+; Nf1−/+) did not show any significant extinction over the course of 1 week, while the other genotypes (Alk+/+, Nf1+/+, Alk−/+; Nf1−/+ and Alk −/−; Nf1−/+) all showed significant extinction with double heterozygotes for Alk and Nf1 (Alk−/+; Nf1−/+), demonstrating fear extinction closest to the wildtype of all Nf1 heterozygotes. 

The implications of the contextual fear learning and extinction results are relevant to the normal function of both Alk and Nf1. Perturbations of this signaling pathway, strikingly, did not alter the acquisition of contextual fear, but strongly reduced the extinction component. Loss of Nf1 and, by implication, increased Alk signaling impaired the ability to suppress a no longer valid association between sound and shock. This plasticity is restored by reducing or removing Alk activity and consistent with a loss of plasticity in cognitive function. 

**Figure 6:**
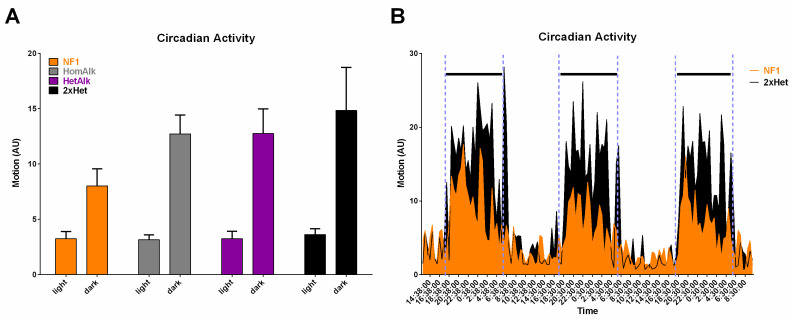
Circadian activity levels in the home cage. (**A**) Nf1 mice (orange bars) showed lower activity levels during the dark phase than Nf1 mice with reduced Alk expression (2 × Het, black bars). The mean activity levels during the light and dark periods are shown, based on circadian activity data from three subsequent 24 h periods. (**B**) Detailed activity levels, averaged over 2 h periods. Nf1 mice (in orange) showed lower activity levels during the dark phase than Nf1 mice with reduced Alk expression (2 × Het, in black). The dark periods are indicated with black lines and are separated by dashed blue vertical lines. AU: arbitrary units. Based on [53].

We also sought to extend phenotypic findings with regard to sleep/diurnal activity from Drosophila to a mammalian model system with the intent of developing therapeutic strategies for this well-documented aspect of human neurofibromatosis (Figure 6). Mice are nocturnal with roughly three-fold more activity in the dark compared to light in laboratory settings. Heterozygous *Nf1* mutant mice show less diurnal variation in activity with increased daytime activity and commensurate decreased reduction in nighttime. The ratio in Nf1 mice is approximately 2:1 compared to 3:1 in wildtype. As in the previously described assays, reduction or removal of Alk function restored a normal diurnal cycle of activity. These results have substantial implications for the therapeutic potential of Alk inhibition in neurofibromatosis in relation to circadian alterations seen in Nf1 patients [36,37,55,56,57,58].

In summary, our initial behavioral studies of Nf1 heterozygous mutant mice confirmed anticipated phenotypes and, most importantly, demonstrated a strong genetic interaction between Alk and Nf1 such that the behavioral phenotypes of Nf1 mutants could be fully or partially rescued by reductions in Alk function. This is consistent with Nf1 acting downstream of Alk as a specific negative regulator in the central nervous system, as had been convincingly demonstrated by two independent groups employing Drosophila as a model system. These results provided a strong justification for pursuing the therapeutic potential of Alk inhibition in treating cognitive and sleep-related phenotypes in Nf1 patients.

## 3. Pharmacologic Inhibition of Alk Rescues a Learning Defect in Nf1 Heterozygous Mice

**Figure 7:**
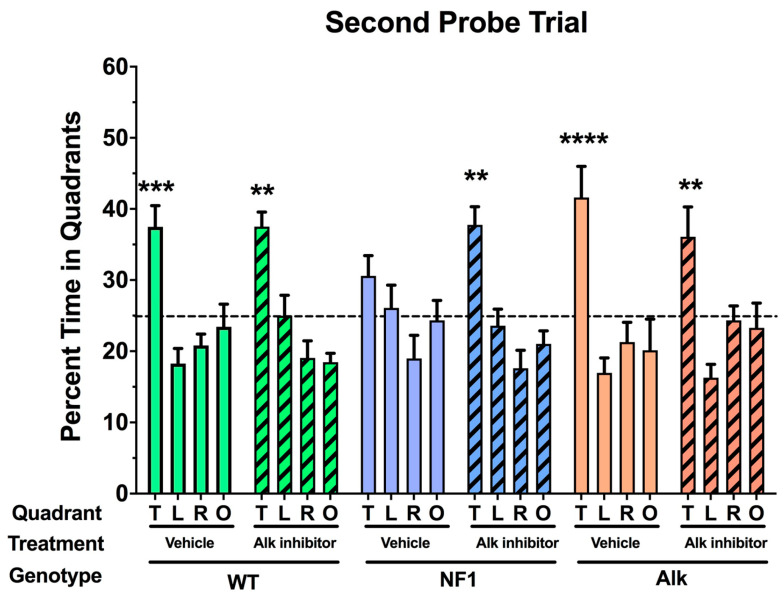
Spatial memory retention in the probe trial (no platform) following training of the second platform location; ** *p* < 0.01, *** *p* < 0.005, **** *p* < 0.001 versus any other quadrant. Adapted based on [64].

Alk had initially been discovered as a human proto-oncogene activated by chromosomal translocation in anaplastic lymphomas [59]. Alk activation was subsequently implicated in non-small cell lung cancer and neuroblastoma as well as other, less common tumors [60]. Small molecule, orally available inhibitors of the Alk kinase domain had been developed to treat these malignancies. The first-generation drugs such as crizotinib did not penetrate the central nervous system, and thus were not appropriate for evaluating the potential of pharmacologic inhibition of Alk [61]. Since cancer does not frequently involve metastatic disease in the central nervous system, newer and more potent Alk inhibitors with CNS activity were developed such as alectinib [62]. Appropriate dosing for our initial studies was a challenge. We were concerned about toxicity and potential off-target effects. In general, the acceptable toxicity of a regime to treat fatal malignancies is higher than the treatment for non-lethal, chronic conditions. With respect to off target effects, we had particular concerns about inhibition of the Alk-related receptor leukocyte tyrosine kinase (Ltk), which we had previously shown to interact antagonistically in Alk-related behavioral phenotypes [63]. We used the lowest effective dose of the Alk inhibitor CH5424802 (alectinib) (1.8 mg/kg) with activity against central nervous system tumors. This dose, which was efficacious, is more than 10-fold lower than the maximal effective dose of (20 mg/kg). We also chose an alternative and more challenging water maze paradigm for testing that we reasoned would be more sensitive to reported Nf1 phenotypes. The alternative learning paradigm employed multiple platform positions in sequential learning trials. This tests cognitive plasticity as well as the ability to learn a new platform location after an initial other location(s) has been acquired. As shown below (Figure 7), we found that Nf1 heterozygotes failed to learn the location of a second location after acquiring the first, but treatment with a small molecule Alk inhibitor corrected this deficiency.

## 4. Development of Alternative Therapeutic Strategies to Inhibit Alk

Our genetic and pharmacologic studies have provided clear evidence of amelioration, and even rescue of neurologic phenotypes associated with Nf1 haploinsufficiency. This approach was motivated by prior studies in Drosophila, demonstrating that reductions in Alk activity interact genetically with Nf1. The strategy can be described as restoration of appropriate Alk signaling in the context of impaired inhibition or hyperactivation due to reduced Nf1 activity. What we hope to achieve is an effective, safe, and specific treatment for patients. It is clear that we have not achieved this goal with the currently available drugs. One aggregate conclusion of our studies is that genetic rescue of Nf1 haploinsufficiency is significantly more robust than any we have demonstrated with long-term alectinib treatment. We speculate that the currently available pharmacologic agents have off-target effects, such as inhibition of the related RTK Ltk, or other pleiotropic effects. With respect to the goal of developing a treatment for neurofibromatosis, our studies may be considered proof-of-concept. In considering drugs developed to treat serious, life-threatening malignancies, as discussed above, tolerance of adverse reactions and toxicities is higher than for treatments of chronic, non-life-threatening conditions such as the neurologic consequences of neurofibromatosis. We have evaluated a long-term treatment with alectinib at two different doses, 3.4 mg/kg and 10 mg/kg, in mice with and without Nf1 heterozygous mutations. We observed that the long-term treatment of Nf1 heterozygous mice with alectinib has some beneficial effects on tests of cognitive behaviors including water maze performance and novel object recognition, but limited any improvement in diurnal activity and anxiety/depressive behavior. The dose required to achieve some of these long-term benefits was 10 mg/kg, although some benefits were also achievable at a lower dose of 3.4 mg/kg. In contrast, a typical dose for treating non-small cell lung cancer in humans with alectinib is approximately 10 mg/kg twice daily. At this dose, a number of adverse reactions have been reported including constipation (34%), fatigue (22%), edema (22%), myalgia (23%), anemia (20%), bradycardia (8.6%), renal impairment (8%), and abnormal liver tests (5%). Even if the incidence of these adverse reactions was reduced to the dose, we anticipate that the long-term treatment would pose challenges for efficacy, safety, and tolerability. We have, therefore, started to investigate alternative, potentially less toxic and more specific strategies to inhibit Alk function.

## 5. Summary and Conclusions

Neurofibromatosis is a disease of haploinsufficiency or inadequate Nf1 function. Groundbreaking studies in Drosophila have clearly established that there is a specific genetic and biochemical interaction between dAlk, a Ras activating RTK, and Nf1, a Ras inactivating Ras-GAP. As demonstrated in multiple contexts, restoring a balance between Ras activation and inactivation via inhibition of Alk or dAlk can ameliorate Nf1 phenotypes. These cumulated investigations provide a unique and exciting opportunity to develop targeted therapy for neurofibromatosis in humans. Multiple strategies for inhibition of gene or protein function have been successfully implemented to address human disease. Pharmacologic or other strategies to enhance gene function have been proven to be much more difficult. Based on these observations, we conclude that a non-toxic, targeted approach to Alk inhibition is the best strategy for developing effective treatments for neurofibromatosis, Nf1. 

In our view, the approach most likely to succeed would entail RNA-based strategies such as RNAi or anti-sense oligonucleotides (ASO), specifically targeting Alk mRNA with enhanced stability and reduced toxicity. To date, four siRNAs have been approved by the FDA for treatment of chronic illnesses such as heritable amyloidosis and hypercholesterolemia. Toxicity and tolerability of these drugs have been well within the acceptable range. The versatility and specificity of RNAi and ASO have been well established in multiple preclinical laboratory studies. We anticipate, however, at least one major obstacle. To date, all approved siRNA drugs have targeted expression of proteins in the liver. None have been successfully deployed to target protein expression in the brain. Based on clear results in Drosophila, that dAlk and dNf1 act in the central nervous system to produce or modify *dNf1* related phenotypes, we anticipate the requirement for therapeutic intervention in the human CNS. There are reports of several techniques that have successfully delivered RNA-based therapeutics to the CNS using targeted nanoparticles, protein RNA conjugates, and targeted exosomes, whose synthesis is directed by the patient’s liver. We hope to explore these options for targeting Alk expression in the CNS as a less toxic, more effective treatment for the behavioral aspects of neurofibromatosis. 

As part of those studies, it is important to consider sex differences. Neuronal dysfunction in Nf1 patients is sex-dependent. Female patients with Nf1-associated optic glioma are twice as likely to undergo brain MRI for visual symptoms and three times more likely to require treatment for visual decline than male patients [65]. In addition, microglia in Nf1 patients show sexually dimorphic cyclic AMP-dependent purinergic impairments. Microglia in males only are being affected [66]. Glioma risk in Nf1 patients is also sex-dependent. Glioma risk is increased in females but reduced in males [67].

Neurological phenotypes might be more pronounced in heterozygous offspring from a maternal compared to a paternal carrier. Our results indicate that the behavioral and cognitive phenotypes and responsiveness to Alk inhibition in heterozygous Nf1 offspring depend on whether there is a maternal compared to a paternal carrier [68]. These parental effects might involve genetic background differences in mitochondria function. As the Nf1 carrier was always on a B6 background and the non-Nf1 carrier was always on a SVJ background, it is conceivable that whether the maternal or paternal is on either background might have contributed to the observed parental carrier effects. Future efforts are warranted to determine whether there might be a parent-of-origin effect in Nf1 patients. If present, this would be of clinical relevance for diagnostic purposes and consideration of treatment strategies. 

Of note, Nf1 is also associated with cardiovascular phenotypes, including systemic and pulmonary hypertension and congenital heart disease [69,70]. Future efforts are warranted to evaluate the therapeutic potential of Alk inhibition for both cardiovascular (CV) and brain dysfunction in Nf1 mice and to determine the underlying mechanisms. 

## Data Availability

The data supporting reported results and related analyses can be found in the referenced primary research papers.

## Supplementary Materials

The following supporting information can be downloaded at: https://www.mdpi.com/article/10.3390/cancers15184579/s1, Table S1: PRISMA 2020 Checklist.
